# Intravitreally Injected Anti-VEGF Antibody Reduces Brown Fat in Neonatal Mice

**DOI:** 10.1371/journal.pone.0134308

**Published:** 2015-07-30

**Authors:** Dong Hyun Jo, Sung Wook Park, Chang Sik Cho, Michael B. Powner, Jin Hyoung Kim, Marcus Fruttiger, Jeong Hun Kim

**Affiliations:** 1 Fight against Angiogenesis-Related Blindness (FARB) Laboratory, Clinical Research Institute, Seoul National University Hospital, Seoul, Republic of Korea; 2 Department of Biomedical Sciences, College of Medicine, Seoul National University, Seoul, Republic of Korea; 3 UCL Institute of Ophthalmology, University College London, London, United Kingdom; 4 Department of Ophthalmology, College of Medicine, Seoul National University, Seoul, Republic of Korea; Faculty of Biology, SPAIN

## Abstract

Anti-vascular endothelial growth factor (VEGF) agents are the mainstay treatment for various angiogenesis-related retinal diseases. Currently, bevacizumab, a recombinant humanized anti-VEGF antibody, is trailed in retinopathy of prematurity, a vasoproliferative retinal disorder in premature infants. However, the risks of systemic complications after intravitreal injection of anti-VEGF antibody in infants are not well understood. In this study, we show that intravitreally injected anti-VEGF antibody is transported into the systemic circulation into the periphery where it reduces brown fat in neonatal C57BL/6 mice. A considerable amount of anti-VEGF antibody was detected in serum after intravitreal injection. Furthermore, in interscapular brown adipose tissue, we found lipid droplet accumulation, decreased VEGF levels, loss of vascular network, and decreased expression of mitochondria-related genes, *Ppargc1a* and *Ucp1*, all of which are characteristics of “whitening” of brown fat. With increasing age and body weight, brown fat restored its morphology and vascularity. Our results show that there is a transient, but significant impact of intravitreally administered anti-VEGF antibody on brown adipose tissue in neonatal mice. We suggest that more attention should be focused on the metabolic and developmental significance of brown adipose tissue in bevacizumab treated retinopathy of prematurity infants.

## Introduction

Anti-vascular endothelial growth factor (VEGF) agents are routinely used in various retinal diseases including age-related macular degeneration and diabetic retinopathy [1,2]. Recently, retinopathy of prematurity (ROP) has also been a target of anti-VEGF antibody, bevacizumab (Genentech) [3,4]. The rationale for the use of bevacizumab is the increased level of VEGF in the eyes of patients with ROP, which is secreted in response to hypoxia in non-neovascularized retina [5]. In ROP, vasoproliferative changes can lead to vision impairment if they are improperly treated or left untreated.

Although intravitreally injected anti-VEGF therapy is thought to be relatively safe in adults [6], studies on the safety in infants are limited [7]. It is not ideal to use therapeutic agents of which safety has not been guaranteed in premature infants. Unfortunately, indiscriminate use of anti-VEGF agents for ROP can induce vision-threatening complications and lasting ocular structural abnormalities [8,9]. The more important issue is that intravitreally injected anti-VEGF agents can be transported into the blood stream and increase the risk of systemic adverse events [10]. Particularly, neonatal Fc receptors in ocular tissues might affect distribution of intravitreally injected anti-VEGF antibody, which has an Fc domain [11,12].

Anti-VEGF antibody in systemic circulation can affect several organs that require VEGF for normal development in developing premature infants. For instance, brown adipose tissue (BAT) is supported by an extensive vasculature which is dependent on VEGF [13]. In this study, we show that intravitreally injected anti-VEGF antibody transiently reduces brown fat in neonatal mice. Since brown fat plays a crucial role in non-shivering thermogenesis and homeostasis regarding energy metabolism [14,15], even transient impacts on these processes give rise to concern in premature infants.

## Materials and Methods

### Oxygen-induced retinopathy (OIR)

C57BL/6 mice were obtained from Central Lab. Animal. OIR was induced in newborn mice as previously described [16,17]. At postnatal day (P)14, we intravitreally injected 1 μL of phosphate-buffered saline (PBS) or anti-mouse VEGF164 antibody (1 μg/eye; cat. no.: AF-493-NA, R&D) into the right eye of mice using NanoFil 10 μL syringe with 35 gauge needle (WPI) after anesthesia using tiletamine plus zolazepam (Zoletil 50, Virbac; 30 mg/Kg) and xylazine (Rompun, Bayer; 10 mg/Kg). At P17, the mice were euthanized by carbon dioxide in deep anesthesia using tiletamine plus zolazepam and xylazine. Then, the enucleated eyes were prepared for immunofluorescent staining of whole mount retinas with Alexa Fluor 594 isolectin GS-IB4 conjugate (5 μg/mL; Invitrogen). The whole mount retinas were viewed with a fluorescence microscope (Eclipse 90i; Nikon). Then, the neovascular tufts were marked and the extent of them were calculated using NIS-Elements AR (v. 3.2; Nikon). The area of neovascular tufts was normalized to the area of whole retina. All animal studies were approved by the Seoul National University Institutional Animal Care and Use Committee and conducted in agreement with the ARVO statement for the use of animals in ophthalmic and vision research.

### Tissue and serum preparation

After euthanasia by carbon dioxide in deep in deep anesthesia using tiletamine plus zolazepam and xylazine, interscapular BAT was meticulously isolated and divided into 3 pieces for paraffin blocks and preparation of protein and RNA, respectively. Before euthanasia, we collected 150~300 μL fresh blood from mice in deep anesthesia by intracardiac puncture and put it into tubes (cat. no.: 365967; BD). After the incubation of 1 hour at room temperature, the tubes were centrifuged at 7,500 rpm at 4°C for 10 minutes. Serum was transferred to microtubes and stored in a deep freezer for further experiments.

### Enzyme-linked immunosorbent assay (ELISA)

Protein was isolated from BAT by centrifugation at 12,000 rpm at 4°C for 20 minutes after homogenization in 200 μL RIPA buffer at 4°C. VEGF levels were measured in diluted serum (1:5) and protein soup from BAT with Mouse VEGF ELISA kit (cat. no.: MMV00; R&D). The amount of anti-VEGF antibody in serum was measured using goat IgG ELISA kit (cat. no.: 7520, Alpha Diagnostic). To estimate the level of anti-VEGF antibody using goat IgG ELISA, we utilized PBS as the control. In the other experiments, we tried to maintain consistency by using PBS as the control. All samples were measured twice (*n* = 3~6).

### Histologic analysis

BAT was fixed in 4% paraformaldehyde at 4°C for 18 hours. Hematoxylin and eosin (H&E)-stained slides from paraffin blocks were evaluated to estimate the number of large lipid droplets (> 50 μm^2^) per each field at magnification x400. Quantification was performed with captured images (4 randomly selected ones per mice) using Image J (v. 1.48v; NIH) after 8-bit conversion, establishment of threshold with an IsoData algorithm, and segmentation with a watershed algorithm (*n* = 3~6).

### Immunofluorescence staining and immunohistochemistry

4-μm-thick paraffin sections were incubated at 4°C for 2 hours and processed with sequential immersion in Xylene Substitute (Thermo) and graded ethyl alcohol solutions. Then, antigen retrieval was performed by immersion of sections in 0.1 M sodium citrate (pH 6.8, Sigma) at 120°C for 10 minutes. After the permeabilization with 0.2% Triton X-100 for 15 minutes, we treated the sections with 1X Universal Blocking Reagent (Biogenex) for 10 minutes to minimize nonspecific binding. For immunofluorescence staining of vessels in BAT, the sections were incubated with Alexa Fluor 594 isolectin GS-IB4 conjugate (4 μg/mL) overnight. Quantification was performed with captured images (4 randomly selected ones per mice) using Image J by the measurement of the proportions of isolectin B4-positive area per image after 8-bit conversion (*n* = 3~6). For immunohistochemistry of UCP1 in BAT, the sections were incubated with primary antibody to UCP1 (1:100, cat. no.: ab10983; abcam) overnight and treated with REAL Detection Systems (Dako) and DAB Kit (Life Technologies) as the manufacturer’s instructions.

### Real-time polymerase chain reaction (PCR)

Total RNA was isolated from BAT using TRI Reagent (Molecular Research Center) according to the manufacturer’s instructions. cDNA was prepared with High Capacity RNA-to-cDNA kit (Life Technologies). Real-time PCR was performed with StepOnePlus Real-Time PCR System (Life Technologies) with TaqMan Fast Advanced Master Mix (Life Technologies) and specific Gene Expression Assays (cat. no.: 4331182; Life Technologies). Product IDs of Gene Expression Assays for genes are as follows: for *Ucp1*, Mm01244861_m1, for *Ppargc1a*, Mm01208835_m1, for *Cox4i1*, Mm01250094_m1, for *Cox4i2*, Mm00446387_m1, for *Gapdh*, Mm99999915_g1, and for *Rn18s*, Mm03928990_g1. All procedures were performed in accordance with the MIQE guidelines.

### Statistics

Differences between control and treatment groups were assessed by 2-tailed unpaired T-test using Prism 5 (GraphPad). The mean ± SEM was shown in figures.

## Results and Discussion

### Intravitreally injected anti-VEGF antibody suppresses retinal neovascularization and is detected in serum

**Fig 1:**
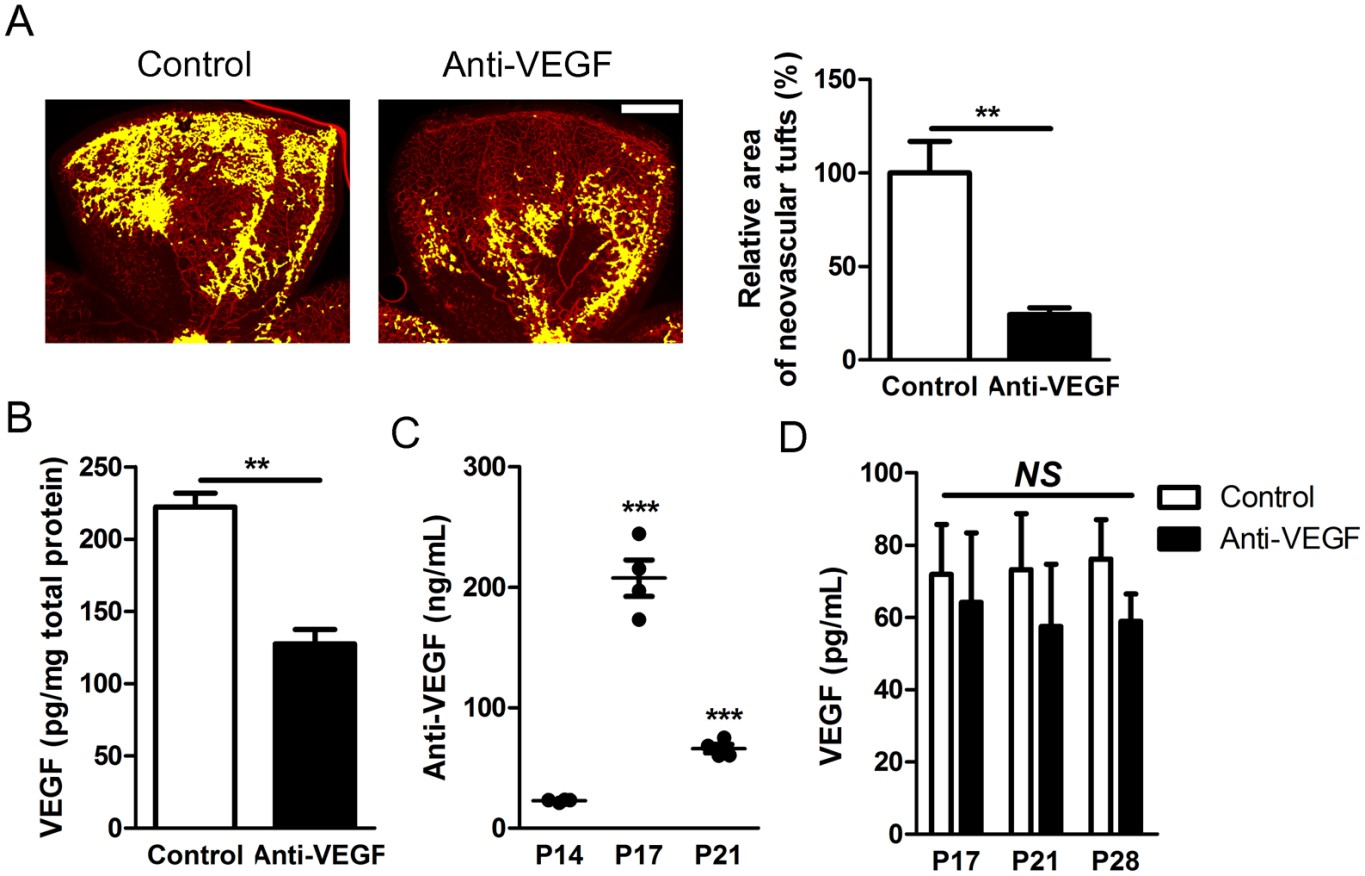
Ocular and systemic consequences of intravitreally injected anti-VEGF antibody. (A) Effects of intravitreally injected anti-VEGF antibody (1 μg/eye) on retinal neovascularization in OIR mice (*n* = 6). Neovascular tufts were highlighted with yellow pseudocolor on representative images of isolectin B4-stained retina. The area of neovascular tufts was normalized to total retinal area; then, the effects of anti-VEGF antibody were quantified and normalized to the control (intravitreal PBS injection). Scale bar, 200 μm. (B) Retinal VEGF concentrations at P17 with intravitreal injection of PBS or anti-VEGF antibody (*n* = 3). The level of VEGF was normalized to total amounts of proteins in the retina. (C) Serum concentrations of anti-VEGF antibody after intravitreal injection at P14, P17, and P21 (*n* = 3–6). (D) Serum VEGF concentrations after intravitreal injection of anti-VEGF antibody at P17, P21, and P28 (*n* = 3–6). Data are presented as mean ± SEM in graphs. Anti-VEGF, anti-VEGF antibody. *NS*, not significant; **, *P* < 0.01; ***, *P* <0.001 (two-tailed, unpaired T-test).

To investigate anti-angiogenic effects of anti-VEGF antibody on retinal neovascularization, we injected anti-mouse VEGF164 antibody (1 μg) into the vitreous cavity of right eyes of OIR mice at P14 (S1 Fig). OIR is a well-established animal model of retinal neovascularization in ROP [16,17]. Retinas of OIR mice demonstrate characteristics observed in those of infants with ROP: retinal hypoxia and retinal neovascularization from existing retinal vasculature. As expected, intravitreally injected anti-VEGF antibody effectively reduced the formation of neovascular tufts in OIR mice (Fig 1A). We also found reduced levels of VEGF in the retina (Fig 1B), which was likely to be the cause of reduced neovascularization.

Next, we measured serum levels of anti-VEGF antibody and VEGF to identify systemic consequences of intravitreally administered anti-VEGF antibody. We estimated the level of anti-VEGF antibody through the measurement of goat IgG. In this regard, we utilized PBS for the control vehicle throughout the whole study. Furthermore, the treatment of anti-VEGF antibody in this study definitely mimicked the clinical use of anti-VEGF antibody in premature infants. Interestingly, the serum concentration of anti-VEGF antibody was found to be 207.6 ± 15.01 ng/mL at P17, 3 days after intravitreal injection (Fig 1C). Based on estimated total blood volume at this age (~0.5 mL), this suggests that around 10% of the injected dosage was still present in the serum. Despite anti-VEGF antibody in systemic circulation, we did not observe significant reduction in serum VEGF levels (Fig 1D).

In the early era of bevacizumab use for infants with ROP, it was believed that bevacizumab is too large to be transported into the blood stream [3,18]. However, there have been reports on unanticipated effects of bevacizumab on the opposite eye after intravitreal injection, suggesting systemic effects [19]. The more direct evidence came from pharmacokinetic studies in laboratory animals and humans [20–22]. In particular, in infants with ROP, bevacizumab was also detected and increased until 2 weeks after intravitreal injection [23]. A possible mechanism might operate through neonatal Fc receptor, which is expressed in ocular tissues and is thought to play an important role in the transportation of IgG into the systemic circulation [11,12].

As in Fig 1D, we did not observe significant changes in the level of serum VEGF although we detected anti-VEGF antibody in the serum. However, we decided to proceed further experiments based on following reasons: 1) systemic levels of VEGF do not reflect tissue levels [10,24]. 2) No reduction or paradoxical increase in total VEGF with bevacizumab in serum is not an unexpected finding [25,26]. 3) Furthermore, we aimed to investigate systemic effects of intravitreally injected anti-VEGF antibody even at the concentrations of no definite change in serum VEGF levels.

### Intravitreally injected anti-VEGF antibody reduces VEGF in BAT and induces “Whitening” of brown fat

**Fig 2:**
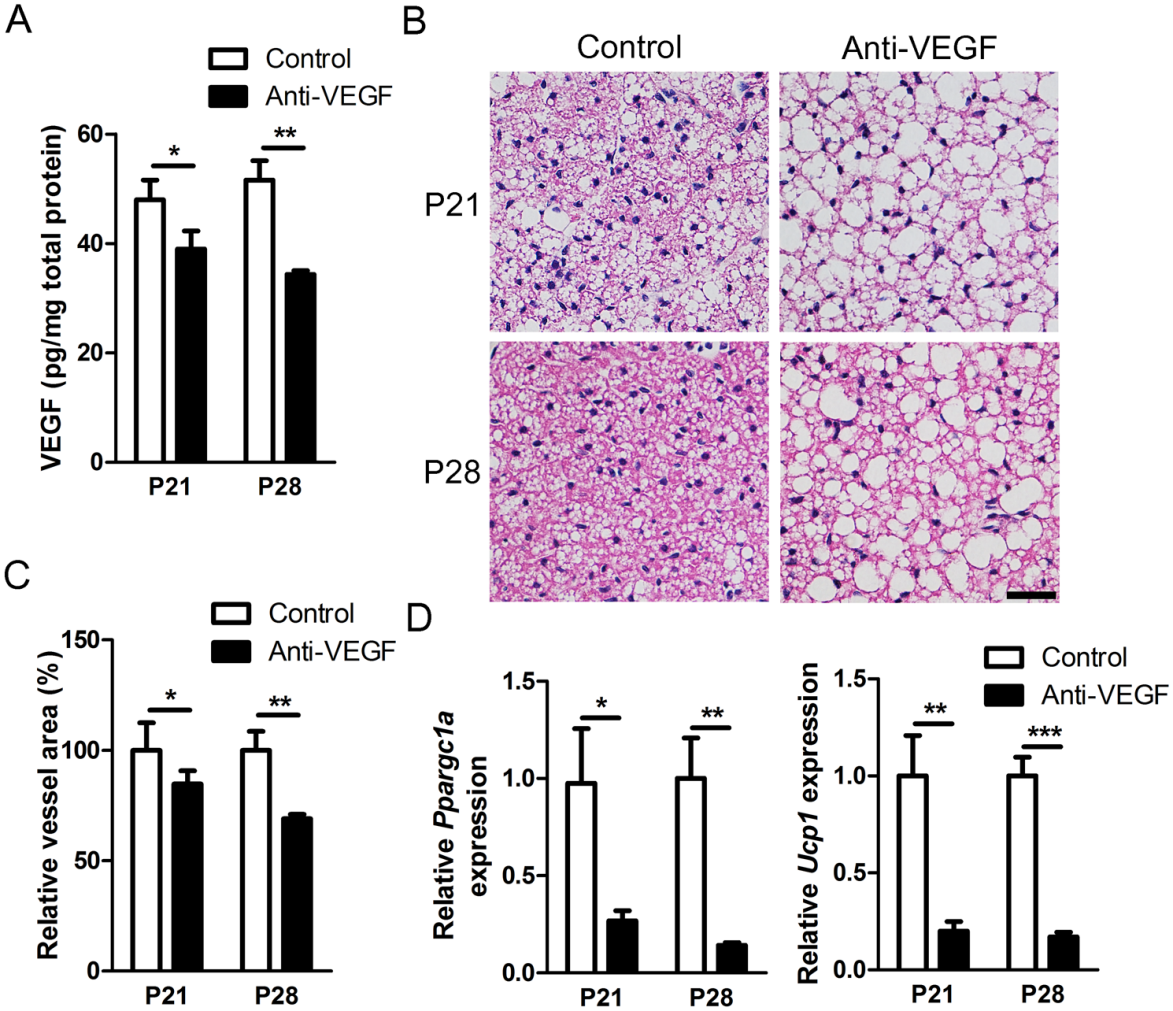
Effects of intravitreally injected anti-VEGF antibody on BAT of neonatal mice. (A) Concentrations of VEGF in interscapular BAT at P21 and P28. The level of VEGF was normalized to total amounts of proteins in BAT (*n* = 3–6). (B) Representative images of H&E staining of interscapular BAT after intravitreal injection of PBS or anti-VEGF antibody show enlarged lipid droplets. Scale bar, 20 μm. (C) Quantitative analyses of vascularity of interscapular BAT based on isolectin B4 staining (*n* = 3–6). The effects of anti-VEGF antibody were quantified and normalized to the control (intravitreal PBS injection). (D) Relative expression of *Ucp1* and *Ppargc1a* in interscapular BAT (*n* = 3–6). Data are presented as mean ± SEM in graphs. Anti-VEGF, anti-VEGF antibody. *, *P* < 0.05; **, *P* < 0.01; ***, *P* <0.001 (two-tailed, unpaired T-test).

Next, we examined VEGF levels in interscapular BAT at P21 and P28, 1 and 2 weeks after intravitreal injection, respectively. It is notable that the size of interscapular BAT were not significantly changed (data not shown). In contrast, at both time points, intravitreally injected anti-VEGF antibody significantly reduces the level of VEGF in BAT (Fig 2A; *P*-value = 0.0317 and 0.0086, respectively). We speculated that the reduction in the level of VEGF might be due to direct scavenging of VEGF by anti-VEGF antibody. Targeted genetic deletion of VEGF in adipose tissue has been previously shown to induce indicators of BAT “whitening”, such as lipid droplet accumulation and mitochondrial dysfunction [15]. In keeping with this, in our study, H&E staining demonstrated that anti-VEGF antibody dramatically increased the number of large lipid droplets (> 50 μm2) in BAT (Fig 2B and S2A Fig). BAT is metabolically active and displays higher vascular density [14]. Moreover, VEGF-A is a key factor in governing angiogenesis in adipose tissue [27]. We therefore evaluated the effects of anti-VEGF antibody on vascular network in BAT and found a clear reduction in vessel density, as demonstrated by isolectin B4 staining (Fig 2C and S2B Fig).

VEGF-dependent angiogenesis is known to determine the thermogenic response of BAT [28,29]. Furthermore, it is linked to the expression of key genes involved in thermogenesis and mitochondrial biogenesis in BAT such as *Ppargc1a* and *Ucp1* [30]. Particularly, heat production of BAT is dependent on the action of UCP1 which is specifically expressed in mitochondria and uncouples electron transport from ATP production [31]. We found that intravitreally injected anti-VEGF antibody significantly reduced the expression of *Ppargc1a* and *Ucp1* at P21 and P28 in BAT (Fig 2D). Furthermore, other genes related to mitochondrial function, *Cox4i1* and *Cox4i2*, were also reduced (S2C Fig). Reduced UCP1 expression was also confirmed at protein level (S2D Fig). Taken together, intravitreally administered anti-VEGF antibody induced “whitening” of BAT as systemic deletion or suppression of VEGF [13].

### Effects of anti-VEGF antibody on BAT are transient

**Fig 3:**
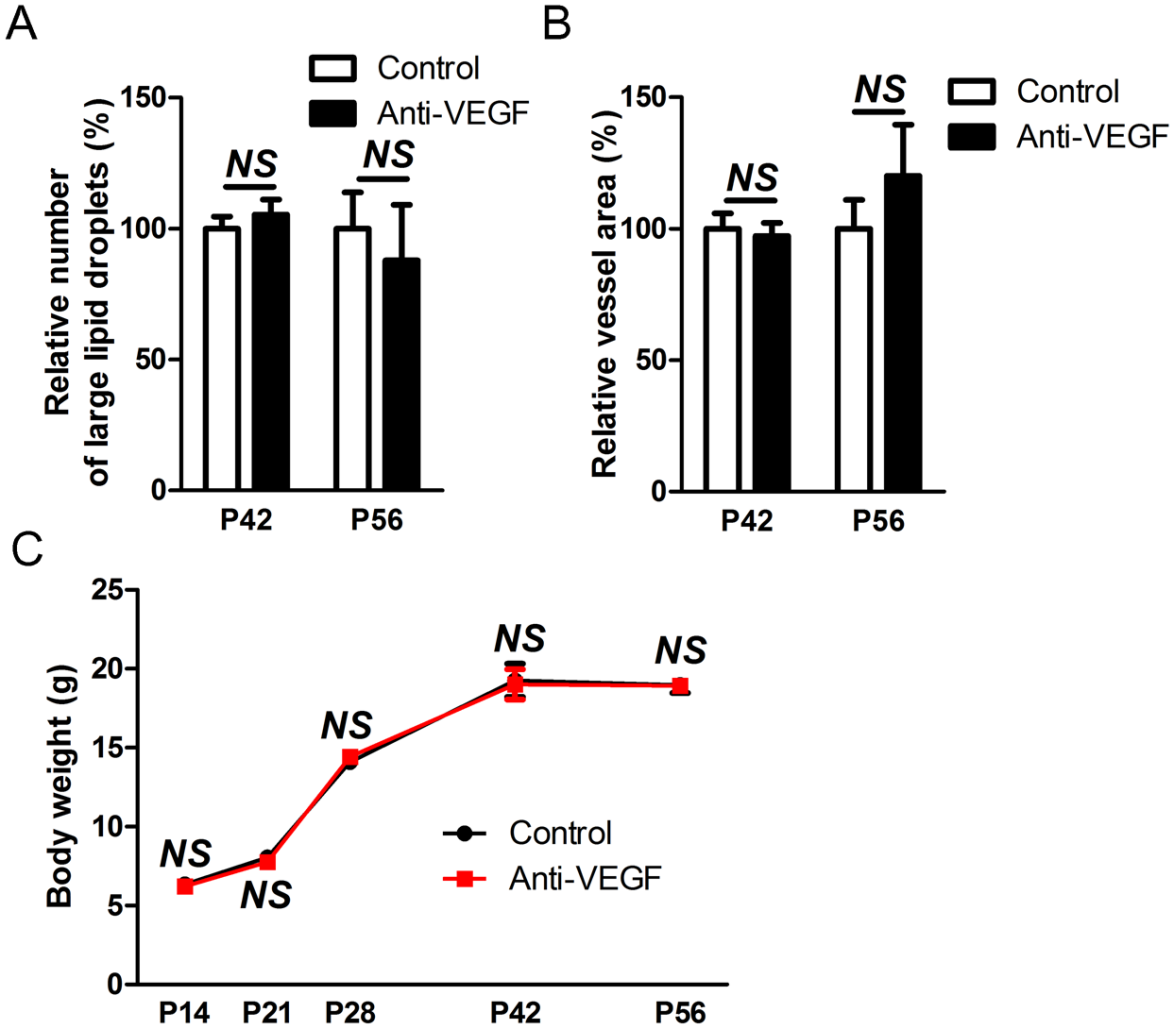
Long-term effects of intravitreally injected anti-VEGF antibody on BAT. (A) Quantitative analyses of the number of large lipid droplets (> 50 μm^2^) per field at x400 magnification (*n* = 3–6). The effects of anti-VEGF antibody were quantitatively analyzed by comparison to the group treated with intravitreal PBS injection as 100%. (B) Quantitative analyses of vascularity of interscapular BAT demonstrated by isolectin B4 staining (*n* = 3–6). The effects of anti-VEGF antibody were quantitatively analyzed by comparison to the group treated with intravitreal PBS injection as 100%. (C) The changes in body weight from P14 to P56. Anti-VEGF, anti-VEGF antibody. *NS*, not significant (two-tailed, unpaired T-test).

To investigate long-term effects of a single intravitreal injection of anti-VEGF antibody on BAT, we examined the morphology and vascularity of BAT at P42 and P56, 4 and 6 weeks after intravitreal injection, respectively. BAT recovered its normal morphology without the distinct large lipid droplets seen at the earlier time points (Fig 3A and S3A Fig). Furthermore, the BAT vascular network also appeared to be comparable to normal controls treated with PBS (Fig 3B). Also, the expression of mitochondria-related genes in BAT of mice treated with anti-VEGF antibody was also comparable to that of mice treated only with PBS at this time point (S3B Fig). These results are consistent with a previous study which demonstrated that the introduction of transgenic VEGF-A could rescue BAT whitening in mice with targeted deletion of *Vegfa* in adipose tissue [15]. Likewise, with decreasing concentrations of serum anti-VEGF antibody (Fig 1C), the effects by neutralization of VEGF in BAT were also diminished.

Despite systemic transportation of intravitreally administered anti-VEGF antibody, bevacizumab is currently in use for the treatment of infants with ROP. There has been so far no definite evidence of serious and life-threatening complications regarding bevacizumab use [7,18]. Similarly, in our study there was no increased mortality in the mouse pups, and we did not observe any permanent changes in body weight between treatment groups during the study period (Fig 3C).

The lack of serious side effects, despite the pronounced effects on BAT, may be due to the transient nature of the changes in BAT. Furthermore, the thermogenic function of BAT is activated under cold exposure or adrenergic stimulus [27,31]. Because our experimental mouse pups (and premature infants) are not normally exposed to excessive cold, it is possible that transient BAT insufficiencies have therefore no further impact. Nevertheless, whitening of BAT might lead to dysfunction in energy and glucose homeostasis [32]. Similarly, VEGF inhibition in BAT of mice resulted in metabolic defects on high-fat diet and decreased insulin sensitivity [15,28]. BAT also modulates other processes of metabolic homeostasis and might have potential involvement in regulation of immune system, cardiovascular system, skeletal mass, and bone metabolism [31–33]. In this context, it is necessary to investigate further implications of the effects of intravitreally injected anti-VEGF antibody. In particular, attention should be paid to developmental problems of bone and skeletal muscle associated with proper BAT function in premature infants. Although BAT possibly demonstrates species-specific distribution and functions, the dependence of proper BAT function on VEGF and its implications should be taken into account [13,14,33].

In conclusion, we have shown that intravitreally injected anti-VEGF antibody can have a transient, but significant impact on BAT in neonatal mice. Reduced VEGF levels in BAT were accompanied by reduced vascular density, increased lipid droplet accumulation, and decreased expression of genes related with function of BAT. Because VEGF also plays an important role in the maintenance and development of other organ systems, systemic effects of intravitreally administered anti-VEGF antibody should be considered in developing infants. Thus, our results provide a further rationale for concerns about the use of bevacizumab in the treatment of ROP, which can be successfully overcome by conventional treatment, laser photocoagulation.

## Supporting Information

S1 Fig Schematic diagram of animal study with OIR mice. From P7 to P12, neonatal mice were exposed to hyperoxia (75% O_2_). At P14, anti-VEGF antibody was injected into the vitreous cavity of right eyes of mice.
(TIF)

S2 Fig Effects of intravitreally injected anti-VEGF antibody on BAT of neonatal mice. (A) Quantitative analyses of the number of large lipid droplets (> 50 μm^2^) per field at x400 magnification (n = 3–6). The effects of anti-VEGF antibody were quantitatively analyzed by comparison to the group treated with intravitreal PBS injection as 100%. (B) Extent of vasculature in BAT according to the treatment with anti-VEGF antibody at P21 and P28. Scale bar, 25 μm. (C) Relative expression of *Cox4i1* in interscapular BAT at P21 and P28 (*n* = 3–6). (D) Relative expression of *Cox4i2* in interscapular BAT at P21 and P28 (*n* = 3–6). (E) Representative images of immunohistochemical staining of UCP1 in interscapular BAT at P28. Scale bar, 50 μm. Data are presented as mean ± SEM in graphs. Anti-VEGF, anti-VEGF antibody. *, *P* < 0.05; **, *P* < 0.01 (two-tailed, unpaired T-test).
(TIF)

S3 Fig Long-term effects of intravitreally injected anti-VEGF antibody on BAT. (A) Representative images of H&E staining of interscapular BAT after intravitreal injection of PBS or anti-VEGF antibody. Scale bar, 20 μm. (B) Relative expression of *Ppargc1a* and *Ucp1* in interscapular BAT at P42 and P56 (*n* = 3–6). Data are presented as mean ± SEM in graphs. Anti-VEGF, anti-VEGF antibody. *NS*, not significant (two-tailed, unpaired T-test).
(TIF)
