# Supplementary figures and images for: Intravitreally Injected Anti-VEGF Antibody Reduces Brown Fat in Neonatal Mice
Source: PLoS One. 2015 Jul 30;10(7):e0134308. doi: 10.1371/journal.pone.0134308 (PMC4520452; doi:10.1371/journal.pone.0134308)

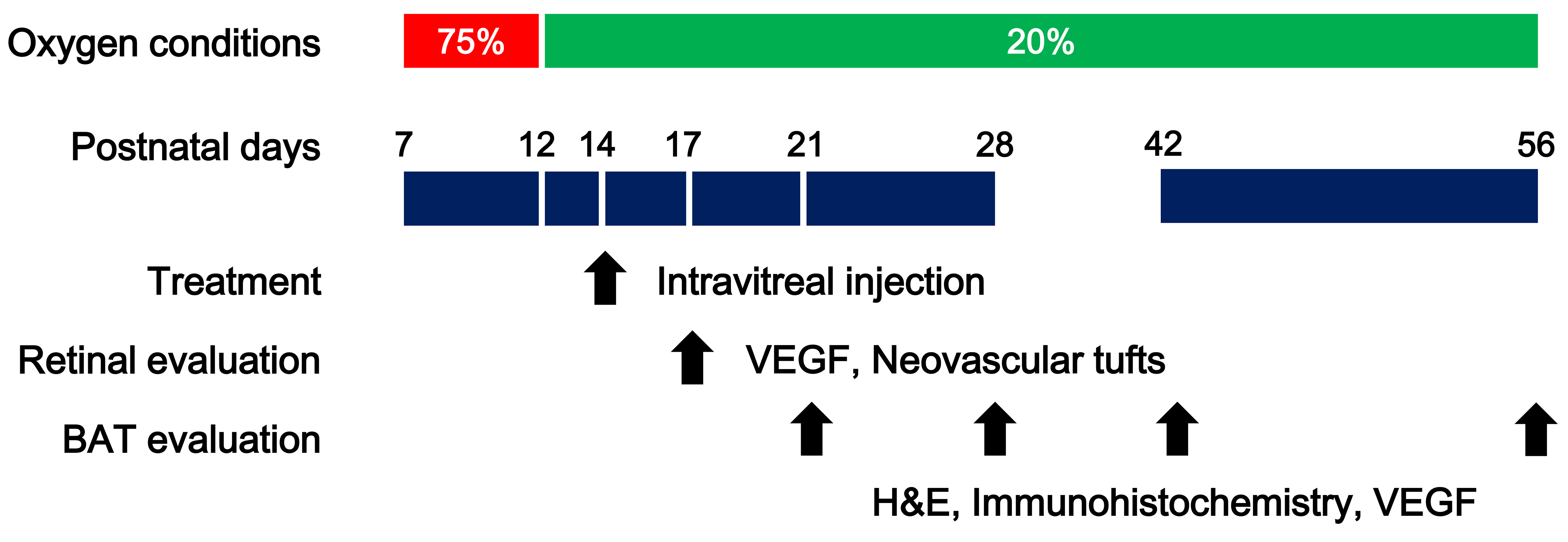

Supplement: S1 Fig — From P7 to P12, neonatal mice were exposed to hyperoxia (75% O2). At P14, anti-VEGF antibody was injected into the vitreous cavity of right eyes of mice. (TIF) [file pone.0134308.s001.tif]

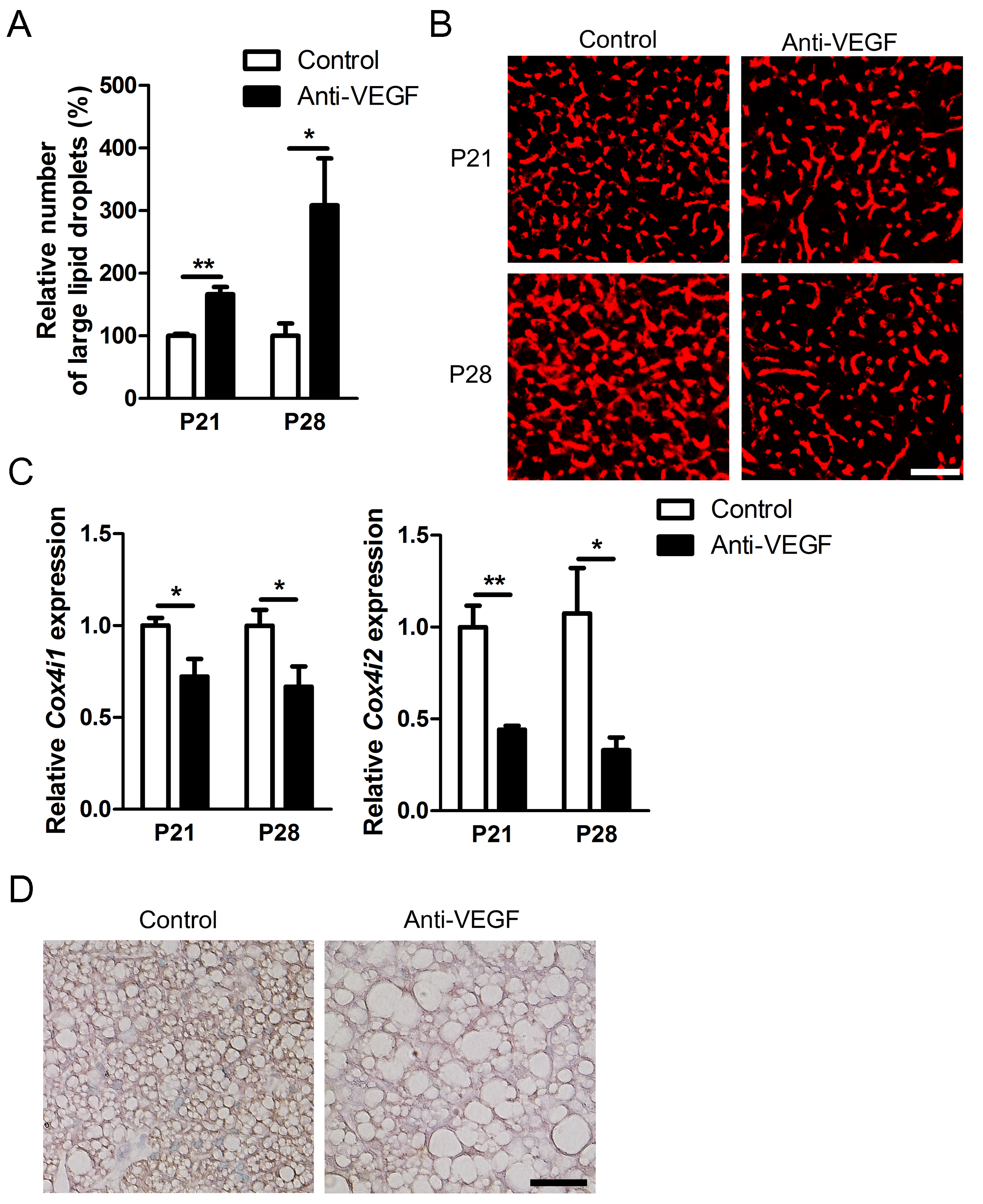

Supplement: S2 Fig — (A) Quantitative analyses of the number of large lipid droplets (> 50 μm2) per field at x400 magnification (n = 3–6). The effects of anti-VEGF antibody were quantitatively analyzed by comparison to the group treated with intravitreal PBS injection as 100%. (B) Extent of vasculature in BAT according to the treatment with anti-VEGF antibody at P21 and P28. Scale bar, 25 μm. (C) Relative expression of Cox4i1 in interscapular BAT at P21 and P28 (n = 3–6). (D) Relative expression of Cox4i2 in interscapular BAT at P21 and P28 (n = 3–6). (E) Representative images of immunohistochemical staining of UCP1 in interscapular BAT at P28. Scale bar, 50 μm. Data are presented as mean ± SEM in graphs. Anti-VEGF, anti-VEGF antibody. *, P < 0.05; **, P < 0.01 (two-tailed, unpaired T-test). (TIF) [file pone.0134308.s002.tif]

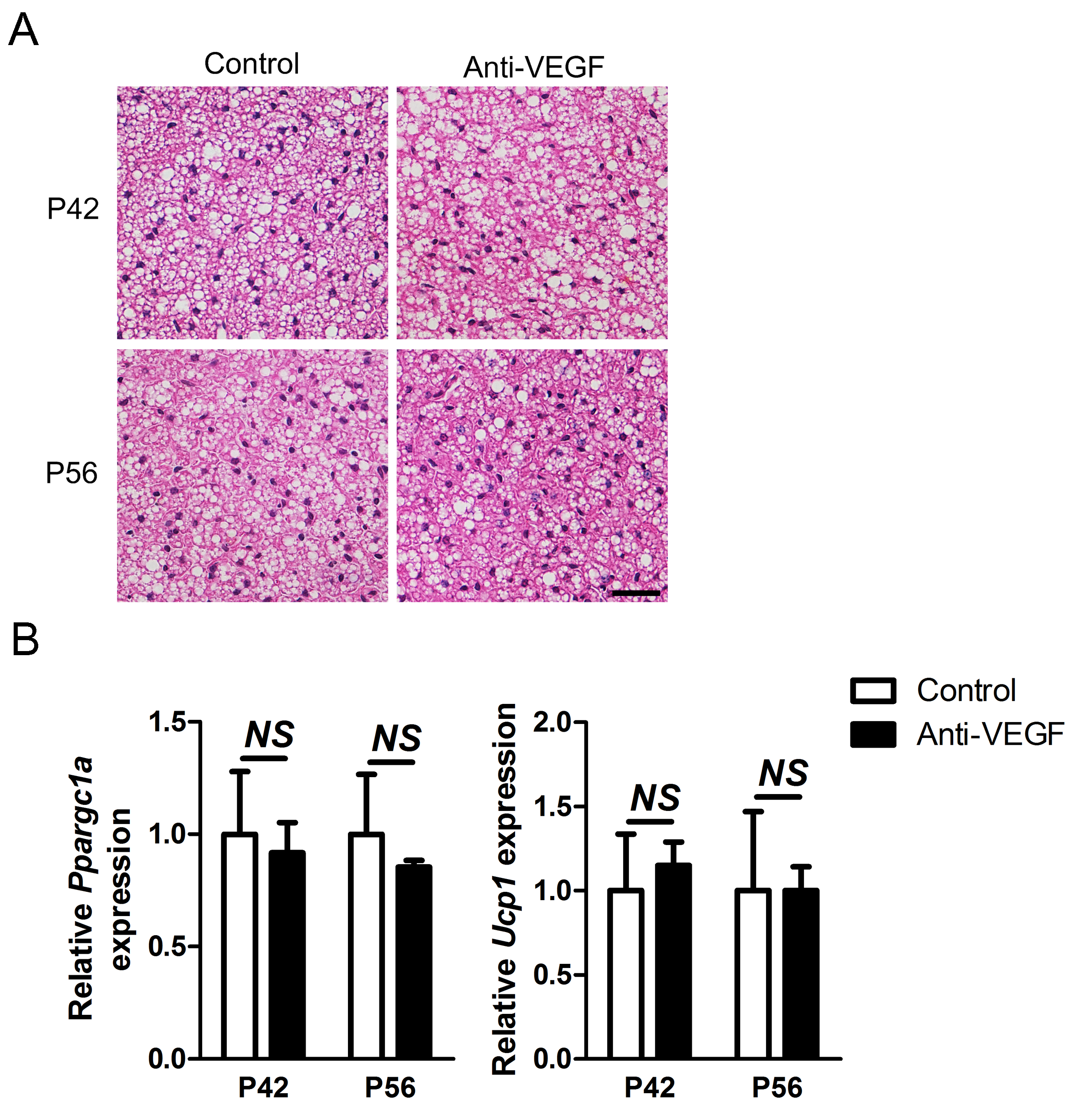

Supplement: S3 Fig — (A) Representative images of H&E staining of interscapular BAT after intravitreal injection of PBS or anti-VEGF antibody. Scale bar, 20 μm. (B) Relative expression of Ppargc1a and Ucp1 in interscapular BAT at P42 and P56 (n = 3–6). Data are presented as mean ± SEM in graphs. Anti-VEGF, anti-VEGF antibody. NS, not significant (two-tailed, unpaired T-test). (TIF) [file pone.0134308.s003.tif]
